# Up-regulation of hexokinase1 in the right ventricle of monocrotaline induced pulmonary hypertension

**DOI:** 10.1186/s12931-014-0119-9

**Published:** 2014-10-08

**Authors:** Wei-hua Zhang, Mei-hong Qiu, Xiao-jian Wang, Kai Sun, Yang Zheng, Zhi-cheng Jing

**Affiliations:** The Center of Cardiovascular Disease, The First Hospital of Jilin University, Changchun, China; Department of Cardiology, Luoyang Central Hospital Affiliated to Zhengzhou University, Luoyang, China; Thrombosis and vascular Medicine Center, College & Chinese Academy of Medical Sciences, State Key Lab of Cardiovascular disease, National Center for Cardiovascular disease, Fu Wai Hospital, Peking Union Medical, 167 Beilishi Road, Beijing, 100037 China

**Keywords:** Pulmonary hypertension, Right heart failure, Glycolysis, Hexokinase 1

## Abstract

**Background:**

Pulmonary arterial hypertension (PAH) is a proliferative arteriopathy associated with a glycolytic shift during heart metabolism. An increase in glycolytic metabolism can be detected in the right ventricle during PAH. Expression levels of glycolysis genes in the right ventricle during glycolysis that occur in monocrotaline (MCT)-induced pulmonary hypertension (PH) remain unknown.

**Methods:**

PH was induced by a single subcutaneous injection of MCT (50 mg/kg) into rats, eventually causing right heart failure. Concurrently, a control group was injected with normal saline. The MCT-PH rats were randomly divided into three groups according to MCT treatment: MCT-2 week, 3 week, and 4 week groups (MCT-2w, 3w, 4w). At the end of the study, hemodynamics and right ventricular hypertrophy were compared among experimental groups. Expression of key glycolytic candidate genes was screened in the right ventricle.

**Results:**

We observed an increase in mean pulmonary arterial pressure, right ventricular systolic pressure and right ventricular hypertrophy index three weeks following MCT injection. Alterations in the morphology and structure of right ventricular myocardial cells, as well as the pulmonary vasculature were observed. Expression of hexokinase 1 (HK1) mRNA began to increase in the right ventricle of the MCT-3w group and MCT-4w group, while the expression of lactate dehydrogenase A (LDHA) was elevated in the right ventricle of the MCT-4w group. Hexokinase 2(HK2), pyruvate dehydrogenase complex α1 (PDHα1), and LDHA mRNA expression showed no changes in the right ventricle. HK1 mRNA expression was further confirmed by HK1 protein expression and immunohistochemical analyses. All findings underlie the glycolytic phenotype in the right ventricle.

**Conclusions:**

There was an increase in the protein and mRNA expression of hexokinase-1 (HK1) three and four weeks after the injection of monocrotaline in the right ventricle, intervention of HK1 may be amenable to therapeutic intervention.

## Background

Pulmonary arterial hypertension (PAH) is a multifactorial and progressive disease characterized by a sustained increase in pulmonary vascular resistance, eventually leading to right heart failure and death [1]. Although the primary insult occurs in the pulmonary vasculature, chronic right ventricle pressure overload stimulates right ventricular hypertrophy and right ventricular failure. The leading cause of death in PAH is right ventricular failure [2,3]. Most researchers and medical professionals agree that the right ventricle (RV) response to pulmonary hypertension (PH) is a major determinant of survival, and a change in myocardial metabolism is a prominent feature of RV dysfunction [4].

Increased right ventricular fluorodeoxyglucose (FDG) uptake has also been described in patients with PAH [4,5], reflecting a shift from oxidative to glycolytic metabolism. Moreover, a glycolytic shift occurs in rodent models of pulmonary hypertension (induced by monocrotaline (MCT) or chronic hypoxia) [6-8] and in models of RV pressure overload induced by pulmonary artery banding [9]. The glycolytic shift was evidenced by an increased uptake of FDG on PET and by direct measurement of metabolism in isolated working RV hearts, and the corrected RV FDG accumulation was decreased after the treatment with epoprostenol in accordance with the degree of reduction in the pulmonary vascular resistance [5]. Furthermore, recent animal data indicated that metabolic derangements in the RV contribute to RV dysfunction [7,9,10]. However, the mechanism of glycolytic metabolism in the RV is still unknown. Whether the glycolytic pathway and glycolysis-related genes participate in PAH has yet to be elucidated; moreover, little is known about the details of the molecular mechanism of PAH development.

In the present study we used MCT-treated rats as an experimental model of PH induced RV failure. MCT is a pyrrolizidine alkaloid, and its bioactive metabolite selectively injures the vascular endothelium of lung vessels. Progressive pulmonary vasculitis leads to increasing vascular resistance and a gradual rise in arterial pressure after a single dose of MCT. The increase in RV afterload induces hypertrophy and eventual failure. The present study investigates the hypothesis that the expression of glycolysis genes in the RV in MCT-PH rats were increased and aims to screen glycolysis relevant genes , including hexokinase1, hexokinase2, and hexokinase3 (HK1, HK2 and HK3), phosphofructokinase (PFK), pyruvate kinase (PK), pyruvate dehydrogenase complex (PDHα1 PDHα1 PDHβ), lactate dehydrogenase (LDHA, LDHB, LDHC and LDHD) and glyceraldehyde-phosphate dehydrogenase (GAPDH; housekeeping gene) using real time polymerase chain reaction (PCR) and validated protein expression.

## Methods and methods

### Animal preparation

Forty-eight adult male Sprague–Dawley rats (body weights, 200 – 220 g) were divided into four groups: control, MCT-2 week, MCT-3 week and MCT-4 week. Control rats received a single subcutaneous injection of saline (n = 12; saline group) and PH was induced by a single subcutaneous injection of monocrotaline (MCT; 50 mg kg-1; Sigma, St. Louis, MO, USA) to cause right heart failure. All experimental procedures were performed in accordance with the Guide for the Care and Use of Laboratory Animals (US National Institutes of Health Publication Number 85–23; revised 1996), and were approved by the Institutional Committee for Use and Care of Laboratory Animals of Tongji University.

### Haemodynamic measurements

After weighing, all rats were anaesthetized with IP 6% chloral hydrate (6 ml/kg), and haemodynamic parameters were measured by a polygraph system (PowerLab 8/30; AD Instruments, Bella Vista, NSW, Australia). Adequate anaesthesia was monitored by the withdrawal response to a paw pinch and respiration monitoring. After a tracheotomy, a polyethylene-50 catheter was inserted *via* the right external jugular vein into the pulmonary artery, The haemodynamic parameter assessment included mean pulmonary arterial pressure (mPAP) and right ventricular systolic pressure (RVSP) at the end of the study.

### Right ventricular hypertrophy and morphometric analyses of pulmonary arteries and right ventricle

The weights of the free-wall of the RV and the left ventricle plus septum (LV+S) were measured separately, and the ratio RV/(LV+S) was calculated as the RV hypertrophy index.

According to the prospective protocol, the lungs and RV were flushed with ice-cold saline following assessment of haemodynamic parameters, and rats were then euthanized prior to morphometric analyses. Sections from the upper left lung tissue and the right ventricular tissues were paraffin-embedded and stained with haematoxylin – eosin (H & E). Arteries of 15–200 μm and right ventricular tissues were evaluated at 400× magnification and analyzed using the Intel Integrated Performance Primitives version 5.0 software (Santa Clara, CA, USA).

### Immunohistochemical analyses

HK1-positive myocardial cells were assessed using HK1 polyclonal antibody staining (1:1000 dilution; Thermo, USA). The number of HK1-positive cells in 10 fields for each section was quantitatively evaluated as a percent of that total of cells at a magnification of 400× in a blind manner [11].

### Total-RNA extraction and first strand cDNA synthesis

Total RNA was isolated from the frozen tissue of right ventricles using the Thizol method [12]. The RNA concentration was determined spectrophotometrically on the basis of absorbance at 260 nm (Beckman, DU800, USA). First strand cDNA was synthesized from total-RNA by using a RT-PCR kit (Takara). The candidate genes were detected using quantitative real-time fluorescent quantitative reverse transcription-polymerase chain reaction (qRT-PCR) in the right ventricular tissue. Samples were added to a microwell plate with TaqMan probes and RT-PCR reagents (Applied Biosystems, Foster City, CA). qRT-PCR was performed with an ABI PRISM 7500 Sequence Detector (Applied Biosystems, Foster City, CA), and primers for rat HK1 HK2 HK3, PFK, PK, PDHα1 PDHα1 PDHβ, LDHA, LDHB, LDHC and LDHD and GAPDH were used. The primers synthesized by ShengGong biological technology in Shanghai. Primer sequences are shown in Table 1.

**Table 1:** **Glycolytic key candidate genes primers and primer sequences used for amplification using real-time PCR**

**primer**	**Primer sequence**		**PCR products**
	**Forward primer (5′-3′)**	**Reverse primer (5′-3′)**	**location size (bp)**
**HK1**	ctgggcttcaccttctcattt	tcgcaagtgggttcttcatac	227
**HK2**	gcagcttctggaggttaagaga	gtctcctttctctgtgccatct	136
**HK3**	gtctgatgatgccttgatggt	cctggagaagtggggatgtag	184
**PFK**	ggatgtctgggaaaatcaaaga	gctcaaaatctgtctggtcctt	141
**PK**	tggtgacagaagtggagcac	gcacaaaggaggcaaagatg	159
**PDHα1**	ccatctcatcactgcctatcg	agcctcctcttcgtcctgtta	98
**PDHα2**	tgtggctctcgcctgtaaata	aaatacctgcccttggttagc	88
**PDHβ**	tagttgcccattcaagaccagt	tccttccacagttacgagatga	167
**LDHA**	caaactgctcatcgtctcaaac	gcaaccacttccaataactctgt	97
**LDHB**	gcataagatggtggtggacag	atccgagagaggtttttcagc	120
**LDHC**	tttactgaagggtctggctga	gacgatttttggagtgctgag	118
**LDHD**	tggcttctgctgtctttccta	gctccctacaactgccttca	100
**GAPDH**	acagcaacagggtggtggac	tttgagggtgcagcgaactt	252

### Western blot analyses

Ventricular tissue homogenates were prepared and sub-jected to electrophoresis on sodium-dodecyl-sulfate polyacrylamide gels, and then transferred onto polyvinylidene fluoride membranes. Membranes were blocked with 5%skimmed milk or 1% bovine serum albumin, and probed with a monoclonal antibody targeting anti-HK1 (1:1000 dilution; Thermo, USA), or anti-GAPDH (1:8000 dilution; ProteinTech, Chicago, IL, USA), followed by the matched secondary antibody (1:2000 dilution; ProteinTech, Chicago, IL, USA). Immunopositive spots were visualized using ECL-Plus™ (Amersham Biosciences, Chalfont St Giles, UK).

### Statistical analyses

Data were analyzed using the Statistical Package for the Social Sciences Ver. 15.0 software (SPSS Inc., Chicago, IL, USA). A one-way ANOVA was used to test for differences among treatment groups, followed by multiple comparisons with the least-significant difference method. The data are reported as mean ± SD of at least three independent experiments. Statistical analysis was performed using unpaired Student’s t-tests to determine the significance between the two means. A probability of *P* < 0.05 was accepted as significant.

## Results

### MCT increased mPAP and RVSP and induced right ventricular hypertrophy

**Figure 1:**
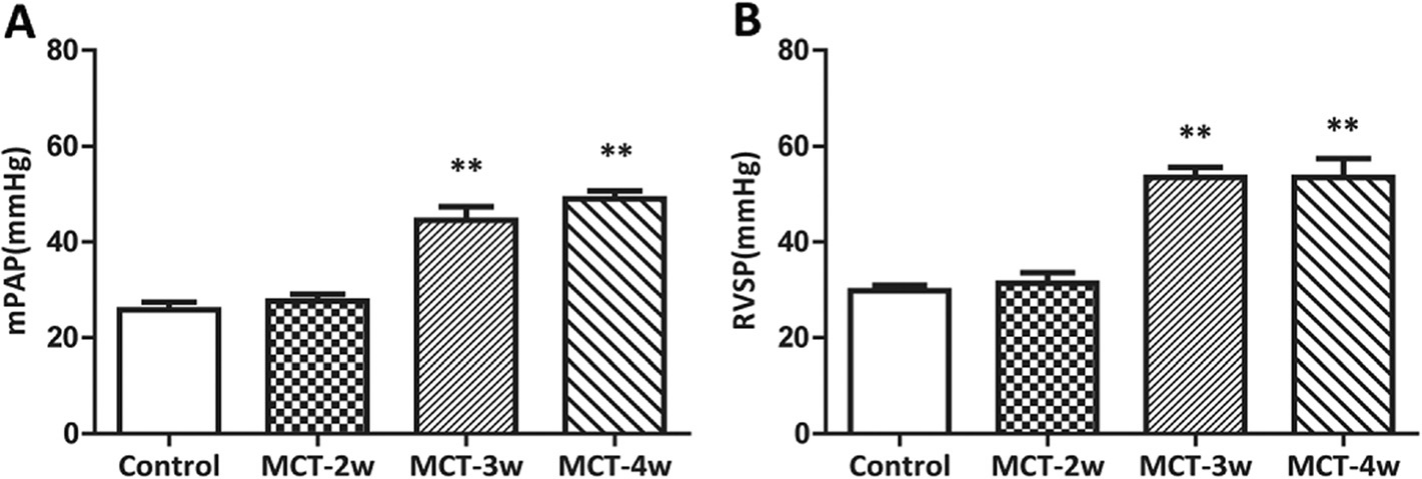
**Monocrotaline (MCT) deteriorateshaemodynamics in rats. A:** Mean pulmonary artery pressure (mPAP); **B:** right ventricular systolic pressure (RVSP); MCT-2w : MCT-2 week group; MCT-3w : MCT-3 week group; MCT-4w : MCT-4 week group; Data shown are means ± SD of 8 –10 rats per group. **P < 0.01 vs. control.

Compared to control group, rats challenged with MCT consistently developed significant PAH with higher mPAP and RVSP in the MCT-3 week and MCT-4 week groups (RVSP:control:27 ± 1 mmHg, MCT-2w:30 ± 2 mmHg; MCT-3w: 52 ± 6 mmHg; MCT-4w: 54 ± 5 mmHg; mPAP: control: 24 ± 1 mmHg; MCT-2w:26 ± 3 mmHg; MCT-3w:45 ± 6 mmHg; MCT-4w:47 ± 5 mmHg, respectively), and the parameters did not rise over time (Figure 1A and B). The ratio of RV free-wall weight to LV + S weight in the MCT group was significantly increased compared with the control group [control group (0.186 ± 0.025), MCT-3 week group (0.285 ± 0.073, *P* < 0.05) and MCT-4 week group (0.329 ± 0.052, *P* < 0.01)]. Moreover, RV hypertrophy positively correlated with MCT treatment time.

### Morphology of right ventricles and pulmonary vasculature

**Figure 2:**
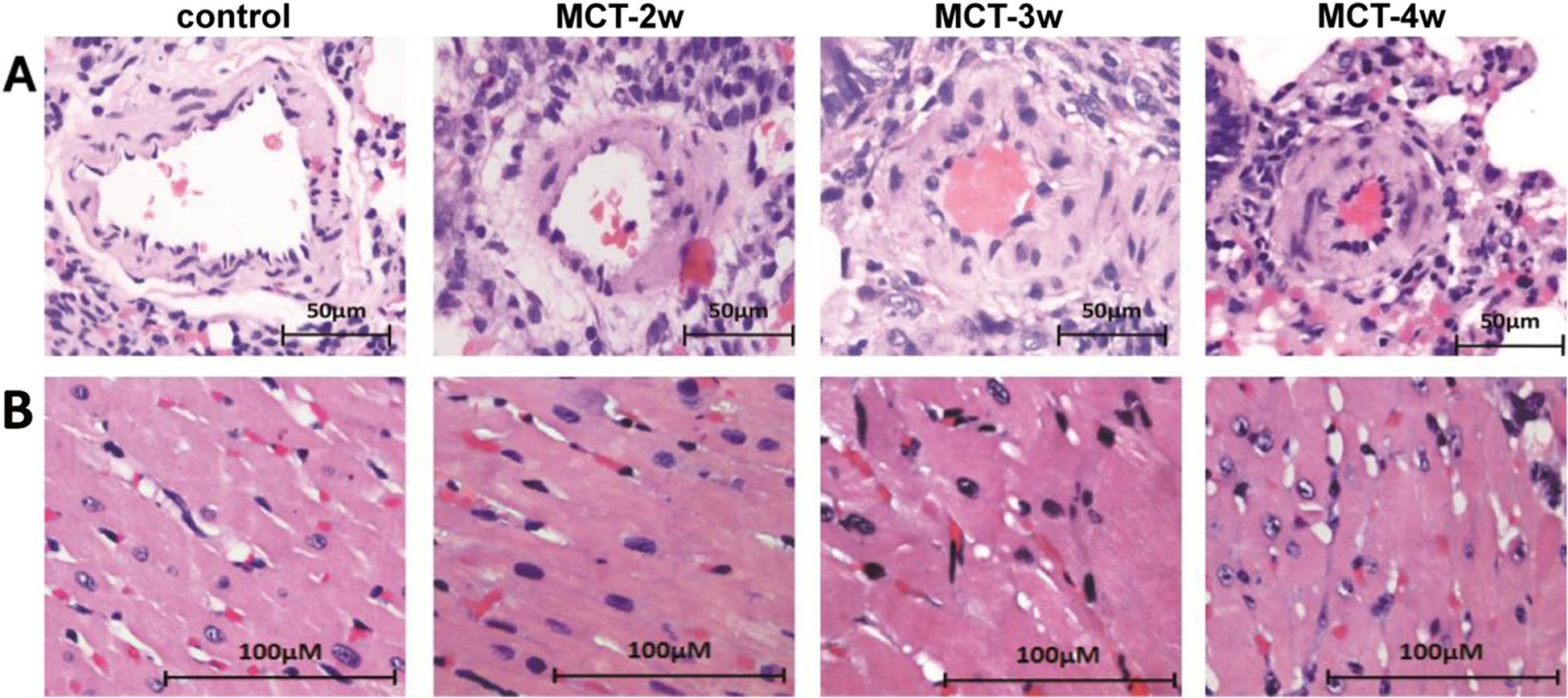
**The change of morphology of lung tissue and right ventricle in rats with MCT treatment time. A:** lung tissue; **B:** right ventricle.

H & E results show that the pulmonary vasculature was thicker following MCT exposure. Remodeled small pulmonary arteries can be observed throughout the lungs (Figure 2A). Also, the myocardial cell nuclei of the control group were in alignment in the RV, muscle fiber directions were consistent, and myocardial cell nuclei were clear. When extending the MCT treatment time (after 3 weeks), the myocardial cells were disordered, muscle fiber direction was unclear, and the myocardial cells sarcoplasm dissolved to increase interstitial fluid (Figure 2B).

### The expression of the genes in the glycolytic pathway in right ventricles

**Figure 3:**
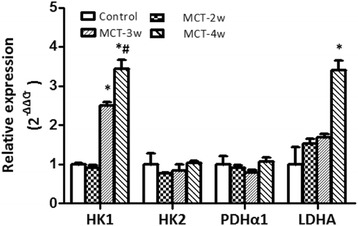
**The expression of the genes in the glycolytic pathway in right ventricles.** HK1: hexokinase 1; HK2: hexokinase 2; LDHA: lactate dehydrogenase A; PDHα1: pyruvate dehydrogenase complex α1. *P < 0.05 vs. controls; #*P* < 0.05 vs. MCT-3w.

Compared with the control group, gene expression of LDHA was up-regulated more than threefold in the MCT-4 week group, while HK1 gene expression raised significantly in the MCT-3 week group (increased ~2.5 fold). Gene expression was clearly higher in the MCT-4 week group (increased more than threefold), and HK2 and PDHα1 did not present any difference in expressed genes (Figure 3), as compared to the control group.

### Expression of HK1 protein in right myocardial cells and the pulmonary vasculature

**Figure 4:**
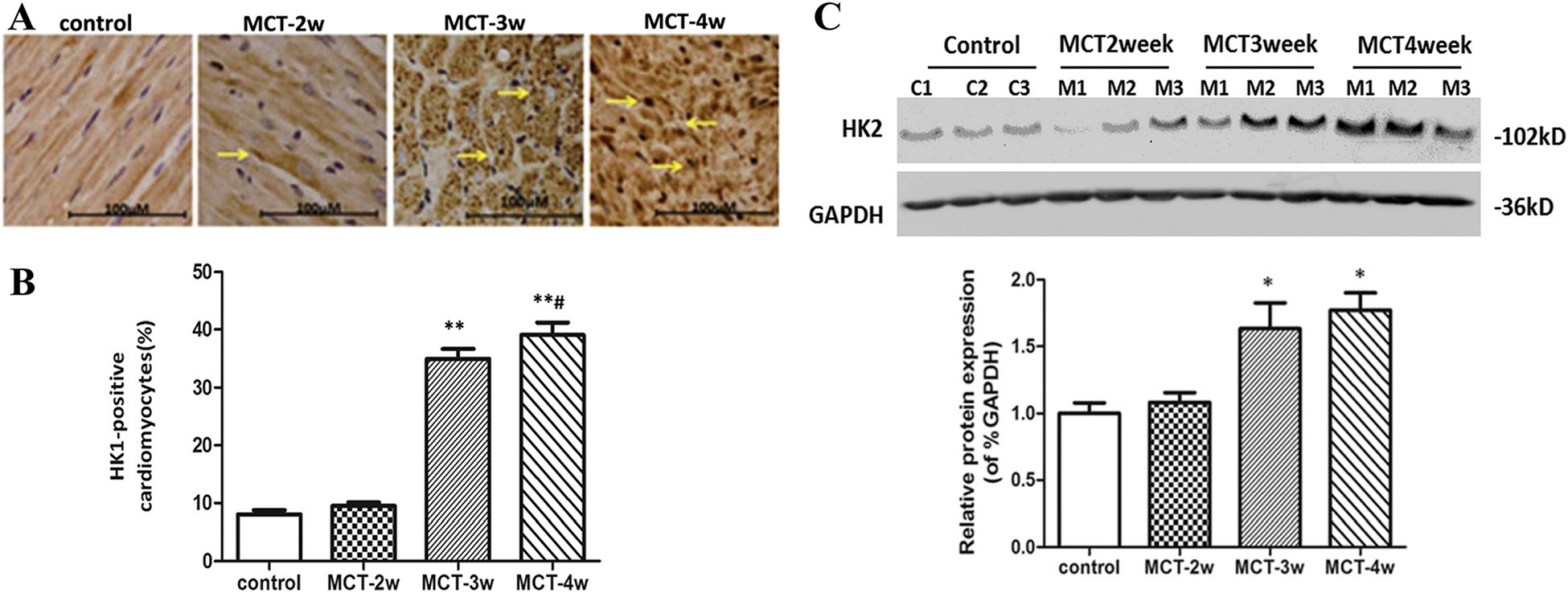
**The expression of HK1 protein is evaluated in right ventricular tissue. A:** The immunohistochemical expression of HK1 in right ventricle; **B:** percentage of HK1-positive myocardial cells in right ventricles; **C:** The expression of HK1 protein in right ventricle. HK1: hexokinase 1; *P < 0.05 vs. controls.

Immunohistochemical results showed that HK1-positive cell expression gradually increased when correlated with MCT treatment time in the RV myocardial cells. The levels of HK1-positive cell expression increased to 33% in the MCT-3w group, and to 39% in the MCT-4w group, compared with the control group (8%) (Figure 4A and B). However, consistent with HK1 gene expression, as compared with the control group, HK1 protein expression was significantly increased in the MCT-3 week (increased ~ 1.6 fold) and MCT-4 week groups (increased ~1.8 fold) in right ventricular tissue (Figure 4C).

## Discussion

It is important that new treatments for PAH address not only the pulmonary vascular pathology, but also the structure and function of RV in PH. For the RV myocardium, it has been shown that myocardial energy substrate utilization is altered in association with the development of pathological forms of ventricular hypertrophy. In this study, we first attempted to verify the activation of these genes relevant to the glycolytic pathway in MCT- PH rats. Moreover, we screened the candidate genes about metabolic pathway in RV, trying to show that some genes are aggravating one of the causes of right heart failure.

Glycolysis involves either glucose or glycogen degrading pyruvate or lactate in the cytoplasm, while releasing a small amount of energy for use. There are three irreversible steps in glycolysis, which are highly exothermic reactions. Our candidate loci included three irreversible rate-limiting enzymes in glycolysis: HK, PFK, PK; the first key enzyme PDH locates the mitochondrial citric acid cycle; and the final product of glycolysis is LDH. Finally, we sought to clarify the possible molecular mechanisms underlying PAH and its association to the glycolytic pathway and glycolysis-related genes.

There was a significant elevation of mPAP and RVSP, and right heart hypertrophy index markedly increased with MCT treatment time. Our study examined different genes in the RV tissues of the glycolytic pathway. We found that both HK1 gene and protein expression were increased, which is one of the three speed limiting enzymes in the glycolytic pathway. HK1 is the first of the glycolytic pathway speed limiting enzymes, and its expression can cause the downstream cascade amplification reaction to provide more ATP to remodel myocardial cells. LDHA gene expression was up-regulated in the right ventricular tissues of the MCT-4 week group, and an increase in LDHA expression occurred after HK1 expression increased. However, Drake JI and colleagues [13] screened and compared different gene expressions between right ventricle hypertrophy and heart failure using gene chip technology, they found HK1 and PFK gene expression was increased in the failing RV compared with the hypertrophied RV.

Glycolytic shift seems to be of significant biological impact in the right ventricle affected by PAH. The reasons of glycolytic pathway activation is the body is in “pseudoanoxic” condition, it may be due to the mitochondrial damage, the expression of superoxide dismutase was lower, it leaded to decreased of hydrogen peroxide in downstream [14]. In patients with PAH, the body feels hypoxic under normal oxygen conditions, leading to the activation of hypoxia-inducible factor α1, which inhibits the mitochondrial electron transport chain and activates pyruvate dehydrogenase kinase (PDK) (which can inhibit PDH), inhibits the Krebs cycle, and reduces ATP production [15]. This may lead to myocardial ischemia and perpetuate right heart failure in PAH. Studies in animal models of PH suggest that dichloroacetate (DCA) (increasing oxidation of glucose) increases cardiac contractility *ex vivo* and *in vivo* [8,16,17]. Restitution of oxidative metabolism with the use of DCA has been shown to be efficient in several animals of PAH. Piao and others have used Fawn-Hooded rats, which develop spontaneous PAH, to demonstrate that oral DCA can increase oxidation of glucose, ATP production, improve right heart function, and increase cardiac output [10].

Overall, in the present study we have demonstrated the ability to screen for candidate genes within the metabolic pathway in the RV. We found that the level of HK1 mRNA was increased in the RV of MCT-PH rats, and HK1 expression correlated with MCT treatment time. In addition, consistent with the expression of HK1 mRNA, the expression of HK1 protein was increased in the RV of MCT-PH rats. However, elucidation of the exact mechanism by which HK1 mRNA and protein were increased in the RV of MCT-PH rats requires further investigation. The aims of future research should include HK1 studies involving other PAH animal models, as well as *in vivo* and *in vitro* studies of related biochemical pathways to evaluate whether HK1 inhibitors can reduce mPAP, RVSP, RVHI and ameliorate PAH. The characterization of the mechanisms underlying the metabolism in PAH, such as cancer, has become emerging targets for novel diagnostic and therapeutic approaches that may have relevance to PH.

## Abbreviations

DCA: Dichloroacetate
FDG: Fluorodeoxyglucose
GAPDH: Glyceraldehyde-phosphate dehydrogenase
H & E: Haematoxylin – eosin
HK1: Hexokinase 1
HK2: Hexokinase 2
HK3: Hexokinase 3
LDH: Dehydrogenase
LV+S: Left ventricle plus septum
MCT: Monocrotaline
mPAP: Mean pulmonary arterial pressure
PAH: Pulmonary arterial hypertension
PAMSC: Pulmonary artery smooth muscle cells
PCR: Polymerase chain reaction
PDH: Pyruvate dehydrogenase complex
PDK: Pyruvate dehydrogenase kinase
PET: Positron emission tomography
PFK: Phosphofructokinase
PK: Pyruvate kinase
qRT-PCR: Quantitative reverse transcription-polymerase chain reaction
RV: Right ventricle
RVSP: Right ventricular systolic pressure
SOD: Superoxide dismutase.
